# Nitinol powders generate from Plasma Rotation Electrode Process provide clean powder for biomedical devices used with suitable size, spheroid surface and pure composition

**DOI:** 10.1038/s41598-018-32101-1

**Published:** 2018-09-13

**Authors:** Tai-I Hsu, Chia-Min Wei, Lu-Dien Wu, Yun-Ping Li, Akihiko Chiba, Meng-Hsiu Tsai

**Affiliations:** 1 Department of health and beauty, Shu Zen College of Medicine and Management, Kaohsiung, 821 Taiwan; 2 Metal Industries Research & Development Centre (MIRDC), Kaohsiung, 811 Taiwan; 3 State Key Lab for Powder Metallurgy, Central South University, Changsha, 980-0812 China; 4 Institute for Materials Research, Tohoku University, Sendai, 980-0812 Japan; 5 Casting section, Metal Industries Research & Development Centre (MIRDC), Kaohsiung, 811 Taiwan

## Abstract

The nickel-titanium alloy (57Ni-43Ti in wt%) was atomized by the plasma rotating electrode process (PREP). The PREP parameters such as plasma arc current, rotating electrode speed with corresponding PREP powder size range in weight percentage analysis, powder morphology and biocapability of cells were studied by scanning electron microscopies, Inductively Coupled Plasma and X-ray diffraction techniques. From the electrode of the produced powders, the composition has no obviously changes. Weight percentage up to 31.8% of the range under 300 μm while the rotation electrode speed increase to 12k rpm. Spherical and flat with smooth surface were observed in different size range. Brittle phase was not observed of XRD data. The nitinol powder has high biocapability with cells showed no cytotoxicity and well cell adhesion in the *in vivo* assay.

## Introduction

NiTi alloy has the characteristic of the shape-memory. It will return to its previous shape from the deformation in some specific temperature. It also contains some significant characteristics, such as super-elastic, anti-abrasive, anti-corrosive, high damping, radiopacity, and generates no effect on MRI. In aerospace engineering application, it has been used to assemble the space station antenna^1^. For medical usage, it is the material for tooth arch wire and coronary stent. However, the application of NiTi alloy is limited by the characteristic of processing difficulty. Some issues come along with the processing, for example, the wastage of cutter when the material is difficult to be processed, the formation of burr is occurred in processing^2^, and surface oxidation happens when welding^3^. Hence, it is usually adopt powder metallurgy to manufacture NiTi alloy, especially to near net shape parts. Powder metallurgy is frequently used in industry, which includes Conventional Sintering (CS)^4^, Self-Propagation High Temperature Synthesis (SHS)^5^, Hot Isostatic Pressing (HIP)^6^, Spark Plasma Sintering (SPS)^7^, Metal Injection Molding (MIM)^8^, and Select Laser Melting (SLM)^9^. There is a key factor how to use NiTi alloy among all these methods that is to fabricate NiTi alloy powder. Usually, NiTi alloy powder is fabricated by gas atomization, however, there are four problems arose in such a processing way^9^: (1) the pollution of crucible could cause the composition control problem; (2) the shape memory ability will be affected by the brittle phase coexistence of Ti_2_Ni, Ti_4_Ni_2_O_x_ and TiNi_3_ instead of the pure NiTi phase in solidification process; (3) gas atomized powders have small adhering satellites which are the small particles attached big powder during manufacturing process; (4) excess oxygen content cause composition changes.

In order to avoid the above bother some problems, plasma rotating electrode process (PREP) is one of the excellent procedures for the production of NiTi alloy powder^10^. PREP has been extensively used in material and metallurgy due to no pollution will be produced as well as those which are generated in gas atomization^11,12^.

The aim of the present work is to link the relationship between process parameter and weight percentage of the different size, the biocapability of NiTi powder so as to biomedical applications.

## Results

### The PREP NiTi powder is spherical in shape without any obviously chemical composition changes from electrode to powders

**Figure 1:**
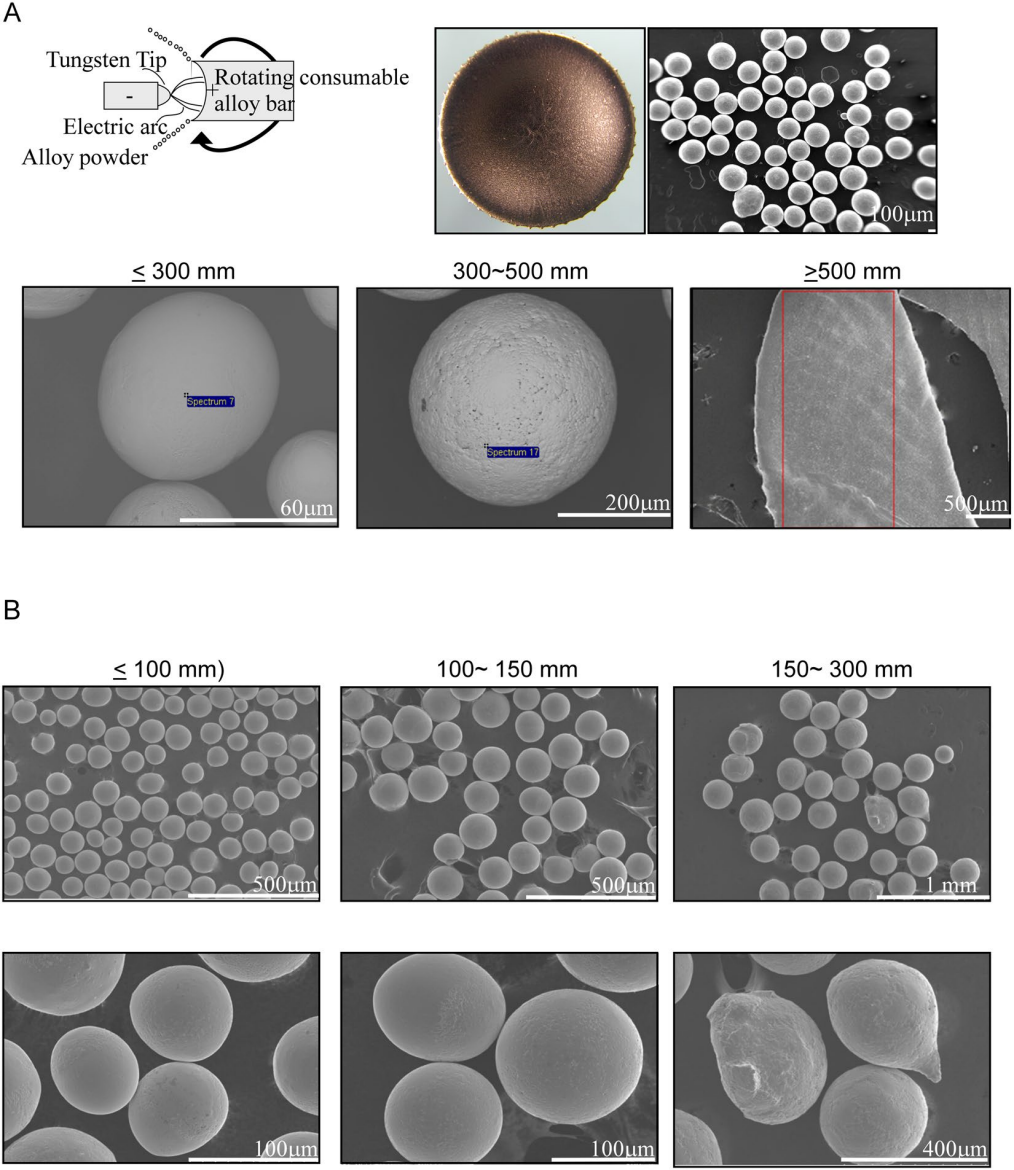
The SEM images showed the powder manufactured by PREP of size distribution pattern from the size smaller than 100 μm to more than 500 μm. (**A**) The schematic diagram modified from ASM book illustrated that the nitinol electrodes and tungsten tip to manufacture the nitinol powder with PREP method. The photography demonstrated the electrode and the powder after PREP manufactured. (**B**) The SEM demonstrated the morphology of powders at different size.

There are some difficulties to produce the NiTi powders. The first step in our project is demonstrated that the powder can be produced from plasma rotating electrode process. The Nitinol rod as electrode was melted by an electric arc, and then becomes metal drops on the influence of the centrifugal force. The nitinol drops were formed at the edge of the electrode in Fig. 1A (left panel). The NiTi powder after cooling was entirely spherical in shape and possesses a smooth surface (Fig. 1A, right panel) due to the surface tension. Table 1 summarizes the chemical composition from electrode with 2 different sizes of powder. The chemical composition analysis showed that the components of electrode compared with powder have no obviously difference. The bulk of the produced particles sieved three sizes in diameter range as ≧500 μm, 300–500 μm, and ≦300 μm were observed (Fig. 1A bottom panel). The size over 500 μm powder shows flat shape (Fig. 1A bottom panel, left side). The flat shape may be formed by colliding with the chamber wall before being solidification at the beginning of the processing. The morphology of different size from 100 to 300 μm shows in Fig. 1B. They are nearly spherical in shape and possesses smooth surface without small adhering satellites under the size of 300 μm.

**Table 1:** ICP chemical analysis of electrode and powder.

Sample	Element Composition (in wt%)		
	Ti	Ni	O
NiTi electrode	43.630	56.128	0.060
NiTi powder over 300 µm in size	43.969	55.763	0.082
NiTi powder under 300 µm in size	44.018	55.687	0.088

### PREP powders are spherical and maintained the NiTi composition

**Figure 2:**
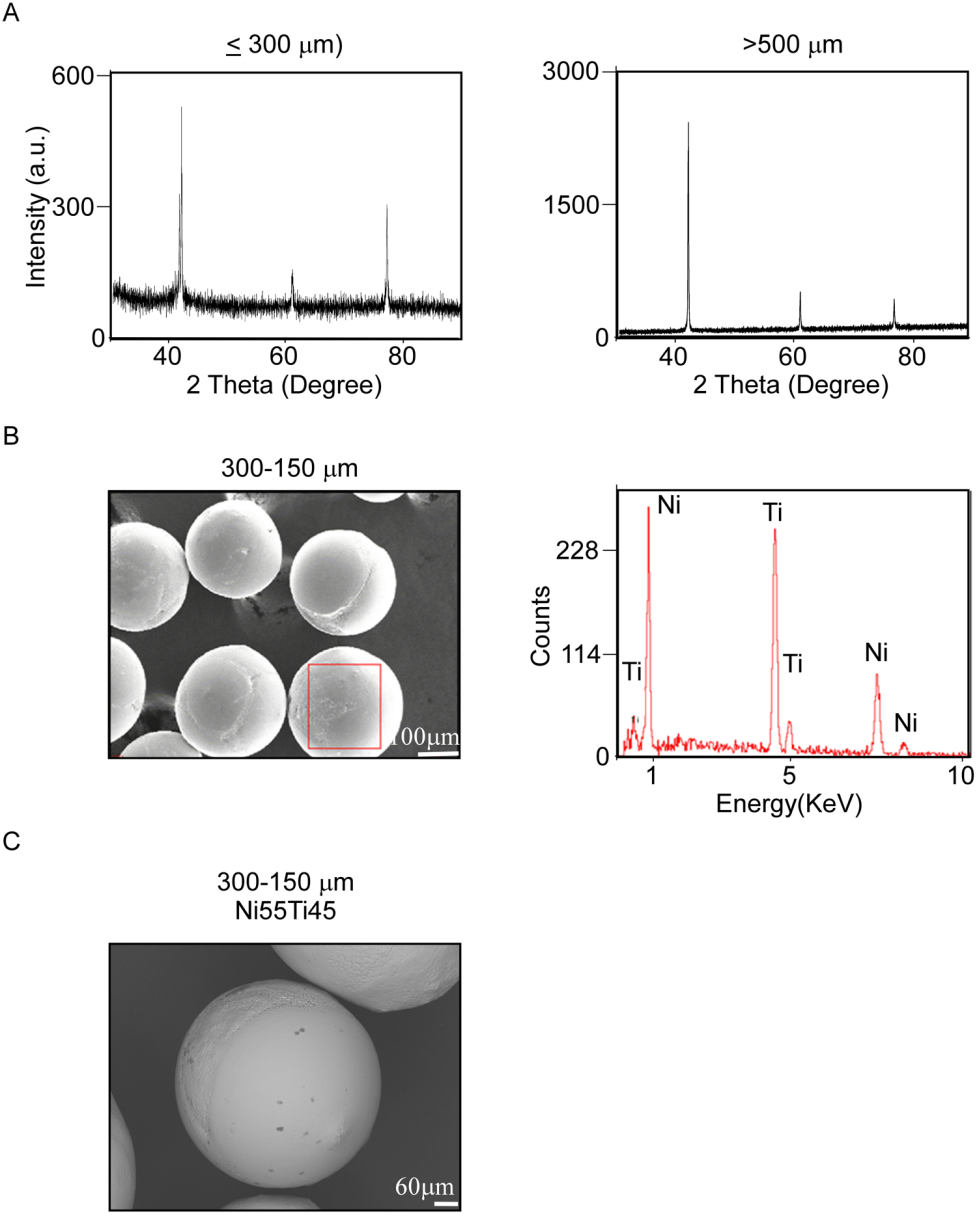
The XRD and EDX examinations confirm Ni-Ti powder component. (**A**) X-ray diffraction analysis of different size powders. The NiTi characteristic peaks (100), (200) and (211) were observed. (**B**) Energy dispersive X-ray spectroscopy result on powder surface (red square) and (**C**) Point EDX result of powder.

The NiTi powders were manufactured from PREP process. However, the element composition is an important issue to verify in order to prove the manufacture method can deal with the NiTi alloy with stable yield and quality. The XRD were performed to assay the different size powder with or without the same crystal structure. The XRD data showed that the powders were the single phase contained only NiTi phase without macro segregation. (Fig. 2A, left panel is the size smaller than 300 μm, left panel is the size more than 500 μm). The EDS assay was performed to confirm the composition of elements. The powders with size from 300–150 μm were used to apply in the EDS experiment (Fig. 2B, left panel). The EDS analysis with region mapping data showed that the 300–150 μm powder with the Ni and Ti peak (Fig. 2B, right panel). The point of EDS spectra also indicated the same results with region mapping (Fig. 2C).

### Increasing electrode rotation speed causes more weight percentage of the small size range

**Figure 3:**
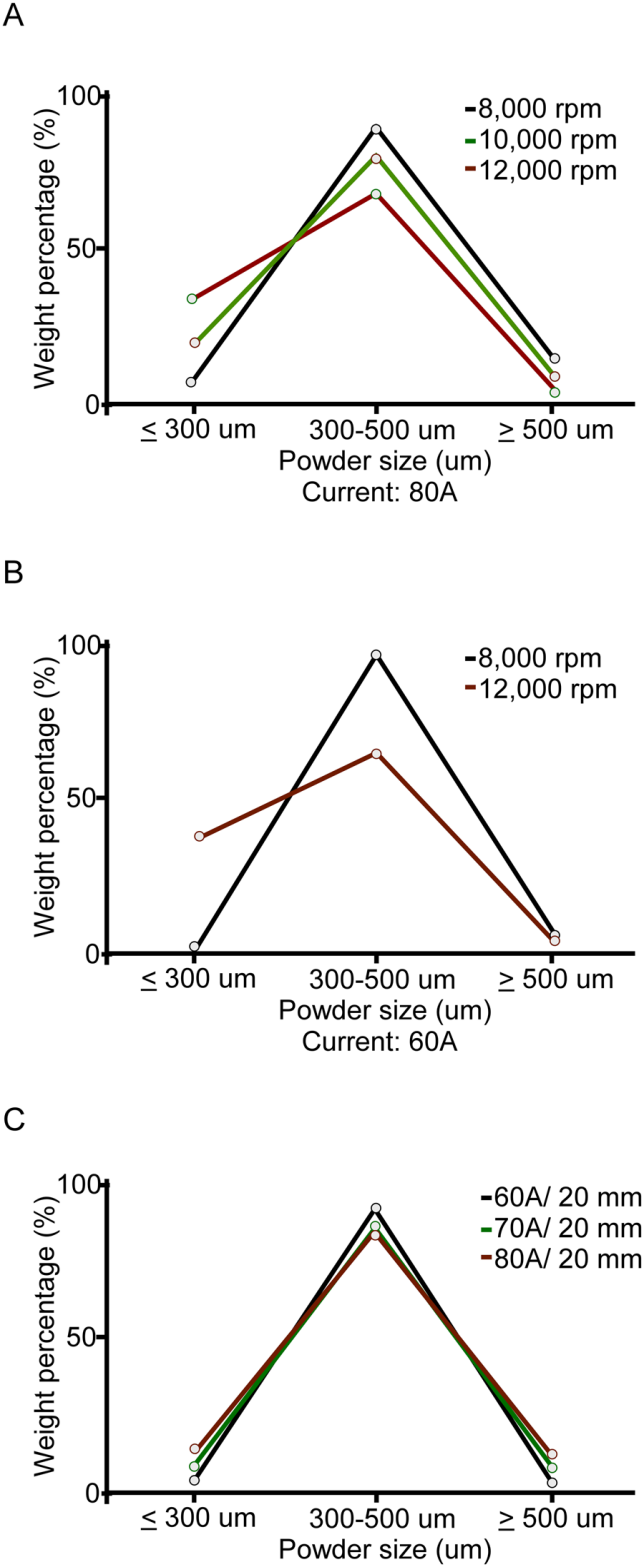
Weight percentage of particular size at different PREP parameters. (**A**) Constant electrode with different arc current. (**B**) Three different rotation electrode speeds on constant arc current 80 A. (**C**) Two different rotation electrode speed on constant arc current 60 A.

The size of powder diameter can influence from electrode diameter and rotation speed. In ASM handbook has been demonstrated that any kinds of alloy with rotating electrode process can use the formula to estimate the particle diameter. The equation listed below it. The PREP powder diameter (d) is determined by

1 $${\rm{d}}={\rm{K}}/{\rm{\omega }}\surd {\rm{D}}$$

where ω (rpm) is the electrode rotation speed, D (mm) is the diameter of the electrode, and K is experience constant (is influenced by the melting rate and material features)^13^. The effects of electrode rotation speed, plasma arc electric current, and electrode diameter on the powder size were studied. Progressive weight percentage analysis of the each range powders shows the relationship between process parameter and their corresponding powder size. The size distribution rate was analyzed to confirm the current and rotation speed can influence the powder size scale. The weight percentage of 300–500 μm is slightly increased (83.4% to 92.4%) and ≧500 μm decreased by lowering arc electric current (from 80 A to 60 A) while ≦300 μm region has no obvious variation (Fig. 3A). This can be understood the arc electrode current is independent with powder size lies on the ≦300 μm region. For lower rotation speed (8k rpm) the weight percentages of ≧500 μm, 300–500 μm, ≦300 μm size region are approximately 12.7%, 83.4%, 3.9% (Fig. 3B). Moderate increase up to 31.8% in ≦300 μm region and decrease to 3.1% in ≧500 μm region are observed for increasing rotation speeds of 12k rpm. Arc electrode current of 60 A, similar tendency was observed in Fig. 3C. This can be understood by the higher rotation speed causing more weight percentage powder in the small size range. Powders lies the 300–500 μm size range are the most abundant in our study.

### Nitinol powder has high biocapability with cells showed no cytotoxicity and well cell adhesion ***in vitro*** assay

**Figure 4:**
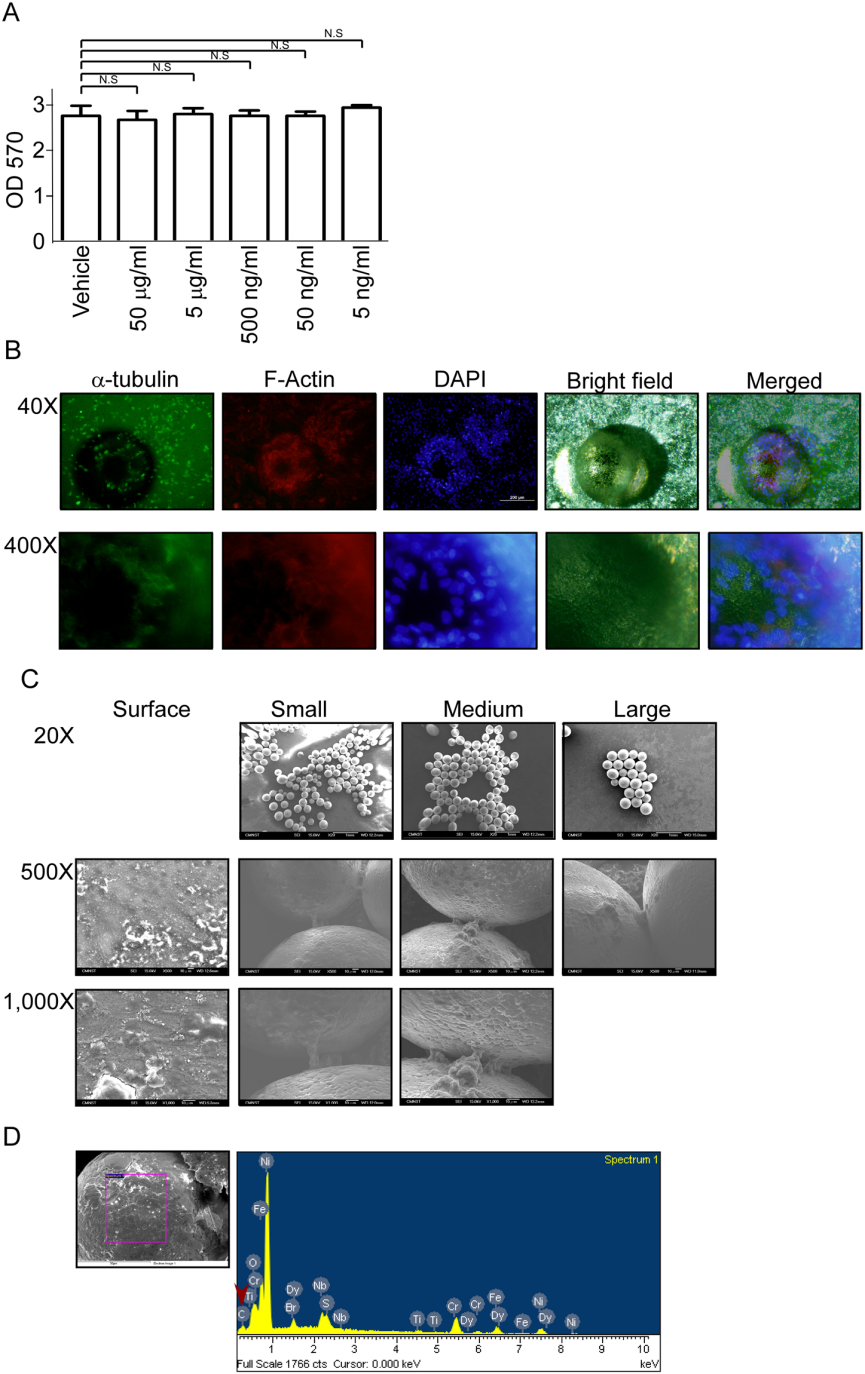
NiTi powder used in the cell showed no cell toxicity. (**A**) The cell viability assay showed that the NiTi powder treated with MG-63 cells did not cause the cell death. (**B**) The red color in IF is F-actin and green is a-tubulin. The IF showed that SaOS2 cells co-cultured with Ni-Ti powder did not affect the lamellipodia and filopodia protrusion. Cells can attach on the powder surface. (**C**) The SEM image demonstrated that cells attach on the powder surface. (**D**) The SEM image indicated the EDS analyzed region. The EDS results there are the cells attach on the surface.

Furthermore, the cell toxicity and viability experiment need to be done in order to identify the NiTi powder suitable for the biomedical devices of implantation type or not. The cytotoxicity need to be evaluated. Cell viability is the first step to validation. MG63 cells derived from osteosarcoma were used to be the cell model. The cytotoxicity assay was performed to examine the material directly contact with cells. The evaluated method is used MTT to detect the mitochondria activity indicated the cell viability. The date from MTT results showed that MG63 co-cultured with NiTi powder with no cell proliferation inhibited from 5 ng/ml to 50μg/ml (Fig. 4A). The biomedical devices has another important issue is the cell can proliferated on the surface or not. Most of the devices need to be contacted with body surface and promoted cell adhesion and proliferated, there are just some material designed for adhesion incapability such as the cardiology devices. Cells can attach, adhesion and mobility on the surface should be depended on the cytoskeleton organization. The cytoskeleton is contained tubulin and actin to organize the cell structure. In our experiment, the red color is F-actin and green color is alpha-tubulin, both of two proteins are the important roles in the cell structure and mobility. The blue color is DAPI to indicate the nucleus in order to identify cell existed or not. The SaOS2 cells were used immunofluorescence (IF) to label the location of protein. The IF data showed that the upper panel is cells distributed in the culture dish and powder surface with well cell structure and organized cytoskeleton (Fig. 4B upper panel, 40X magnification). The lower panel was demonstrated that the cells can attach and adherent on the powder surface (Fig. 4B lower panel, 400X magnification). The IF data showed that cells can attach on the powder surface, the scanning electron microscopy were used to significantly observe the cell structure. The SEM data showed that cells cultured on the glass slides were extended at 1^st^ column in the Fig. 4C. The 2^nd^, 3^rd^ and 4^th^ column were the cell cultured with small, medium and large size powder. The images showed that the cells with powder can adherent on the surface with filopodia and lamellipodia protrusion cross over the gap between powders in all kinds of sizes. From the images, the data can identify Ni-Ti powder with well cell adherent ability can support cell growth. To ensure the surface material is cells. The EDS mapping were be performed to examine the surface of material is living organic. The left image in Fig. 4D was to point out the analyzed region. The right panel is the result of EDS which showed that there is carbon content on the examination region. The NiTi powders were dissolved in the medium to examine the elution with metal can influence the cell growth or not. In the Table 2, the data showed that different sizes of powder did not detect the Ti existed, but the Ni had detected in the medium. Above the results, the data have been identified the NiTi powder with no cytotoxicity and well surface property for cell growth.

**Table 2:** Element analysis of medium from Ni-Ti powder solved.

Sample/element composition (ppm)	Ni	Ti
Ni-Ti small	0.299 ± 0.38 (0.0275–0.5705)	N.D
Ni-Ti medium	0.3629 ± 0.47 (0.0285–0.6973)	N.D
Ni-Ti large	1.10915 ± 1.55 (0.0106–2.2077)	N.D

## Discussion

The *in vitro* assay demonstrated that NiTi powder in cells culture with no growth inhibition. The inductively coupled plasma (ICP) analysis of the medium also showed that the separating rate in the different size particle with low separating rate of Ni and Ti (Table 2). Although the NiTi alloy with high stability property, low degradation rate and low oxidation rate, the element of tolerance rate is still need to be concerned. The ICP data showed that the concentration of Ni is increased with particle size. The data indicated that the NiTi alloy powder has the potential be released and separated after implanted in living body. In our ICP data is followed the ISO10993 guideline to extract the element. The alloy in the MEM medium with 8 hours shaking for 5 days to take the sample in order to perform ICP assay. In future, the segregation in micro scale and the *in vivo* assay with long term study should be performed to identify the devices from NiTi powder manufactured.

In the powder production, there are many process including gas atomization, water atomization, plasma atomization, electrode induction melting gas atomization, centrifugal atomization, plasma spheroidisation and plasma rotating electrode process. The most common method to produce metal powder is the gas atomization. The advantages are powder most are spherical, heat sizes range are flexibility (from 5 kg to 3000 kg), powder size from 0–500 micron and the alloys including Ni, Co, Fe, Ti and Al can be used. The disadvantages are the powder can be asymmetric, the satellites showed in the product, the yield of 20–150 micron are produced from 10 to 50%. However, in our project we want to produce the clean powder for the biomedical device used. The PREP is the better way to get the clean high purity powder, small batch, spherical in shape, extremely high active alloy. The disadvantage is the feedstock bar contacted plasma with bar melted to decrease the yield. In the commercial process, the cost needs to be concerned. The cost of gas atomization is more cheap than PREP. The gas atomization is widely used in the powder produce can reduce the devices cost. PREP cost high is caused low yield.

NiTi alloy powder with low oxygen content and spherical surface morphology has been fabricated by PREP in our experiment. The effects generated by parameters of PREP on powder size are proposed. It shows that increased electrode speed and size are required to obtain refined powder. In addition, it is necessary to consider the dynamic balance issue when increasing the electrode speed. In our study, we have shown a good improvement can be achieved by reducing the length of the electrode bar when dealing with the deviation phenomenon. In future, we will provide better improvement by modifying the mechanism of the equipment.

## Materials and Methods

### NiTi Alloy Electrode Bar

NiTi alloy similar in composition to commercial NiTi (57 Ni-43Ti in weight %) was prepared from nickel, titanium, iron, chromium, copper, nobelium. These elements were melted in a vacuum induction melting (VIM) and vacuum arc re-melting (VAR). The electrode of PREP process was obtained from the ingots and forge to Bars of 20 mm in diameter and 200 mm in length.

### NiTi Alloy Powder Manufactured by PREP

The employed PREP equipment is produced by Nissin Giken Corporation in Japan. It has a stainless steel atomization tank of 1500 mm in diameter with 300 mm in width. Argon gas flow plasma discharge of 6–9 kVA power under10^−3^Pa was produced between the tungsten-tip (cathode) and the nitinol rod of 20 mm in diameter and 200–240 mm in length (anode), starting from the edge of the spinning rod. Rotation speed range is from 8,000~12,000 rpm and the electric current ranges from 60 to 80 A.

Nitinol powders were sieved in three sizes range of about ≧500 μm, 300~500 μm, and ≦300 μm. To get the weight percentage of the each size range nitinol powders at different PREP parameters, three size ranges were weighted. Weight percentage analysis was the weight ratio between the total PREP powder and particular size at every experimental. The microstructure was studied by optical microscopy, and by scanning electron microscopy (SEM) in JEOL JSM-7100 equipment. The powders for SEM were pasted in the carbon tap. In addition, X-ray diffraction (XRD) was carried out in Bruker D8 equipment employing Cu Kα radiation.

### Cell viability assay

Cells were counted and seeded 5000 cells per well in the 96 wells plate. NiTi powder was put in the basal medium MEM (Minimum Essential Media, Invitrogen) to be the stock solution for added into 96 well plates to generate the different concentration contain 5 ng/ml to 50ug/ml in the culture plate. After 24 hours, the NiTi powder were added into the plate and incubated for 72 hours. MTT drug (3-(4,5-dimethyl-2-thiazolyl)-2,5-diphenyl 2 tetrazolium bromide, purchased from invitrogen)were added into plate (the ratio of MTT and medium is 1:5) and incubated 1–4 hours to ferment the tetrazolium bromide to formazan which is the crystal with blue violet color^14–16^. The medium contained in the plate were aspirated and added DMSO (Dimethyl sulfoxide, Sigma) to solve the crystal in order to measure the wavelength of 570 nm. The plates were be measured by Multi-Mode Microplate Reader.

### Immunofluorescence (IF)

Cell were maintained in the culture dish and subcultured every 2–3 days for experiment. The cells were subcultured and seeded on the glass cover slides with NiTi powder contained. The concentration of NiTi powder is 50 ug/ml to performed the IF assay. The cells were cocultured with powder and incubated in 37 °C with 5% CO_2_ chamber for 72 hours. After 3 days, the cells were fixed with 4% paraformahehyde for 15 minutes. The slides were washed with PBS (Phosphate buffered saline) and immersed in the blocking solution for 1 hour. The primary antibodies (F-actin with phallodin and a-tubulin were purchased from Abcam) were added for 16–18 hours in 4 °C. The slides were washed and added the secondary antibody (Invitrogen) to amplify the signal for 1 hour. The slides were washed and with DAPI (2-(4-amidinophenyl)-1H -indole-6-carboxamidine, sigma) to stain the nucleus. The slides were washed mounted for the observation in the microscopy (Nikon). The slides were excited at 280, 488 and 543 nm for emission at 320, 520 and 580 nm to collect the blue, green and red color. The magnification folds were 40 and 400 to observe the cytoskeleton and structure.

### Scanning field microscopy (SEM)

The cells were cultured and fixed with the same methods of IF. After fixation, the slides were used the critical point drying to make samples dry out. Before the SEM observation, the slides were coated with platinum to increase the conductivity for better image quality. The slides were observed to gain the image and analyzed to define the elements by SEM (JEOL 7100).

### Energy-dispersive X-ray spectroscopy

The electron microscopy was performed on the powder surface and with cells using the microscopy equipped with electron gun and X-ray detector combined X-ray analysis system for analysis all elements. The elements were mapped by electron beam and recorded by computer control with counts of X-ray combined with X-ray energies of element of studied. The recording maps obtained from the pixels with time as 512 × 512 pixels and the dwell time of each pixel was typically 51 gs. The probe current was used in the powder and with cells. The candidate elements from X-ray energy window were selected to analysis Ni and Ti elements. The data were analyzed of the number of x-ray to quantify the counts of millioles per kilogram.

### Inductively Coupled Plasma (ICP)

The chemical composition analysis was performed using Inductively Coupled Plasma (ICP) method. 100–240 mg of NiTi powders were mixed 30 mL of HNO_2_ and 10 mL of HF in a Teflon beaker and then heated in order to dissolve the powder in the solution. The solution was dried and the residue was in 40 mL of Aqua regia (HCl:HNO_3_ = 3:1). The final solution was diluted in a volume mask with Mili-Q water, and the concentrations of Ni, Ti, Fe, Cu, Co, and Nb were measured using a Agilent 720-ES ICP instrument.
